# Macular Pigment Optical Density Levels Reflect Retinal Remodeling in Lamellar Macular Holes

**DOI:** 10.1167/iovs.67.4.58

**Published:** 2026-04-27

**Authors:** Alberto Quarta, Corina De Santis Ciacci, Maria Ludovica Ruggeri, Luca Cerino, Lisa Toto, Paolo Carpineto, Rodolfo Mastropasqua

**Affiliations:** 1 Department of Neurosciences, Imaging and Clinical Sciences, University “G. d'Annunzio” Chieti-Pescara, Chieti, Italy; 2 Azienda USL-IRCCS di Reggio Emilia, Reggio Emilia, Italy; 3 Department of Medical, Oral and Biotechnological Sciences, University “G. d'Annunzio” Chieti-Pescara, Chieti, Italy

**Keywords:** macular pigment optical density, lutein, lamellar macular hole, Müller cell cone

## Abstract

**Purpose:**

To quantify macular pigment optical density (MPOD) in eyes with lamellar macular holes (LMHs) diagnosed according to contemporary optical coherence tomography (OCT) criteria and to evaluate its relationship with structural and functional parameters.

**Methods:**

This prospective observational study screened 66 patients with unilateral LMH. All participants underwent comprehensive ophthalmic examination, spectral-domain OCT, and MPOD assessment using heterochromatic flicker photometry. Central foveal thickness (CFT), best-corrected visual acuity (BCVA) and OCT features were recorded. Paired inter-eye comparisons and linear mixed-effects regression analyses were performed.

**Results:**

Forty-eight patients (48 LMH eyes and 48 contralateral healthy fellow eyes) were included. CFT was significantly reduced in LMH eyes compared with fellow eyes (177.2 ± 13.6 µm vs. 251.9 ± 5.9 µm; *P* < 0.0001). MPOD was lower in LMH eyes than in fellow eyes (0.508 ± 0.096 vs. 0.583 ± 0.097; *P* < 0.0001), and BCVA was worse (*P* < 0.0001). In multivariable analysis restricted to LMH eyes, MPOD was independently associated with age (β = −0.010 per year; 95% CI, −0.018 to −0.003; *P* = 0.0085) and BCVA (β = −0.494 per logMAR; 95% CI, −0.910 to −0.079; *P* = 0.0207), but not with CFT (*P* = 0.1438). In eyes with marked foveal thinning (CFT < 180 µm; *n* = 26), MPOD was reduced (*P* = 0.0065).

**Conclusions:**

MPOD is reduced in eyes with OCT-defined LMH and is associated with BCVA. As MP is preferentially localized within the Müller cell cone, altered MPOD may reflect foveal remodeling or MP redistribution related to EP.

Lamellar macular holes (LMHs) were originally described by Gass in 1975 as a partial-thickness foveal defect characterized by an oval configuration and the apparent persistence of residual retinal tissue at the fovea.^1^ More recently, an international panel of vitreoretinal experts proposed a refined optical coherence tomography (OCT)-based consensus definition restricting the diagnosis of LMH to lesions demonstrating apparent degenerative loss of inner retinal tissue.^2^^,^^3^ Under this framework, the entity previously termed “tractional LMH” was reclassified as epiretinal membrane (ERM) foveoschisis, reflecting the absence of tissue loss and the presence of mechanical displacement of the inner retina.^2^^,^^3^ Epiretinal proliferation (EP), foveal bump, and disruption of the ellipsoid zone (EZ) were considered supportive, but not mandatory, features for the diagnosis of true LMH.^3^

Attempts to further characterize LMH using short-wavelength blue fundus autofluorescence were based on the observation that the normal foveal autofluorescence is attenuated by macular pigment (MP), composed primarily of the xanthophyll carotenoids lutein, zeaxanthin and meso-zeaxanthin.^4^ MPs are highly concentrated at the foveal center and foveal region and are localized within the Müller cell cone (MCC), a MC phenotype highly involved in foveal structural support and MP storage, as well as within photoreceptor axons in Henle's fiber layer and the outer nuclear layer.^5^^–^^10^ Disruption of the foveal architecture, particularly involving the MCC may therefore unmask foveal autofluorescence, providing an indirect indicator of tissue loss and assisting in the differentiation of LMH from macular pseudohole when OCT findings are equivocal.^4^ Given the central role of MCCs in foveal stability and MP localization, alterations in MP distribution or density may reflect underlying MCC remodeling in LMH.

Despite growing interest in the structural features of LMH, quantitative data on macular pigment optical density (MPOD) in eyes with OCT-defined LMH remain limited. Heterochromatic flicker photometry (HFP) provides a validated psychophysical method for estimating MPOD and offers a potential means to investigate MP changes associated with foveal tissue loss.^11^^,^^12^ We hypothesize that LMH-associated remodeling of the inner foveal architecture, particularly involving MCCs where the MP is preferentially localized, may lead to alterations in MPOD. The aim of this study was to assess MPOD measured by HFP in eyes with LMH diagnosed according to contemporary OCT criteria and to explore its relationship with structural and functional metrics.

## Methods

### Study Design and Participants

This cross-sectional observational study screened 66 Caucasian patients and included 48 eyes from 48 consecutive subjects with unilateral LMH examined at the Ophthalmology Clinic at the University “G. d'Annunzio” Chieti-Pescara (Chieti, Italy) between April 1, 2020, and October 31, 2022. The study protocol adhered to the tenets of the Declaration of Helsinki and was approved by the local Institutional Review Board (LED31316). Written informed consent was obtained from all participants prior to enrollment.

Eligible participants were required to have a diagnosis of unilateral LMH established on spectral-domain OCT (SD-OCT) based on the following criteria^2^^,^^3^: (1) irregular foveal contour; (2) presence of a foveal cavity with undermined edges documented in at least two horizontal, vertical, or oblique scans separated by approximately 240 µm; (3) at least one additional sign of foveal tissue loss, such as a pseudo-operculum or central or paracentral foveal thinning; and (4) absence of a full-thickness foveal defect. LMH eyes were further characterized according to the presence or absence of the following optional OCT features:^2^^,^^3^ (1) EZ disruption; (2) EP; and (3) foveal bumps (FBs). The contralateral fellow eyes from the same participants, confirmed to be free of vitreomacular interface abnormalities, were used as an internal control group. All patients were locals consuming a Mediterranean diet defined as a Mediterranean Diet Adherence Screener (MEDAS) score ≥ 9.^13^^,^^14^

Exclusion criteria were applied to both eyes of each participant and included (1) previous ocular trauma or intraocular surgery; (2) retinal disease other than LMH in the study eye; (3) axial length greater than 26.0 mm; (4) amblyopia; (5) glaucoma; (6) current or previous uveitis; (7) media opacities, including corneal abnormalities, cataract, or vitreous opacities, precluding reliable imaging or MPOD measurement; and (8) presence in the fellow eye of disruption or changes in inner retinal layers, disruption or changes of outer retinal bands, evidence of foveal contour irregularity,^15^^,^^16^ cavitation, EP, ERM, or vitreomacular traction. Lens opacities were graded according to the Lens Opacities Classification System III (LOCS III).^17^ Eyes with nuclear opalescence or nuclear color greater than grade 2 or cortical or posterior subcapsular cataract greater than grade 2 were excluded.

### Study Population and Procedures

All participants underwent a comprehensive ophthalmic evaluation including best-corrected visual acuity (BCVA) assessment using Snellen charts, slit-lamp biomicroscopy of the anterior segment, and dilated fundus examination. Snellen visual acuity values were converted to logarithm of the minimum angle of resolution (logMAR) units for statistical analysis. Pupillary dilation was achieved using topical tropicamide (10 mg/mL). Retinal imaging was performed using SD-OCT (SPECTRALIS HRA+OCT; Heidelberg Engineering, Heidelberg, Germany), with a multicolor confocal scanning laser ophthalmoscope (cSLO) and fundus autofluorescence (FAF) that supported the LMH diagnosis. The imaging protocol included a macular volume scan comprised of 49 horizontal B-scans spaced 11 µm apart and covering an area of 20° horizontally by 15° vertically, along with a set of 24 high-resolution radial scans centered on the fovea. Automated real-time image averaging was used to enhance image quality, with up to 51 frames (25–51) averaged per B-scan. FastTrac motion correction software was enabled during acquisition. Scans with poor image quality, significant motion artifacts, or inadequate foveal centration were excluded and repeated when possible. The diagnosis of LMH was initially assessed by dilated fundus examination and subsequently confirmed using multimodal imaging. All multimodal imaging was performed after MPOD measurement.

### MPOD Measurement

MPOD was estimated using HFP as previously described.^9^^,^^11^^,^^18^^–^^20^ Briefly, MPOD was measured using the MP Screener II (Elektron Technology, Cambridge, UK), which provides MPOD values on a scale from 0 to 1, with lower values indicating greater transmission of blue light to the foveal cones. Before MPOD testing, all participants underwent a fixation assessment using the built-in fixation task of the device, as recommended in prior HFP studies.^12^^,^^18^ Only eyes demonstrating stable and central fixation, defined by the ability to maintain steady fixation on the target throughout the pretest and test phases without loss of alignment or repeated fixation breaks, were included in the analysis. Eyes with unstable fixation or inability to reliably perform the flicker detection task were excluded.^18^ During MPOD measurement, the device presented two superimposed flickering stimuli, blue (465 nm) and green (530 nm) initially at a frequency above the critical flicker fusion threshold and progressively reduced at a rate of 6 Hz/s. Participants were instructed to press a response button as soon as flicker was perceived. Measurements were obtained in the detailed test mode, which assesses both the central retina (1° circular target centered on the fovea) and a peripheral reference location, with fixation directed to a 1.75° red spot positioned at 8° nasal eccentricity. The instrument calculated MPOD as the logarithmic ratio of blue light luminance between the central and peripheral measurements. Each MPOD assessment was performed twice, with a 30-minute interval between tests to improve repeatability. Only results classified as “accept” by the internal reliability algorithm of the instrument were included in the analysis; tests flagged as “caution” or “reject” were repeated or excluded. MPOD measurement was performed as the first step in the multimodal imaging protocol.

### OCT Analysis

**Figure 1.**
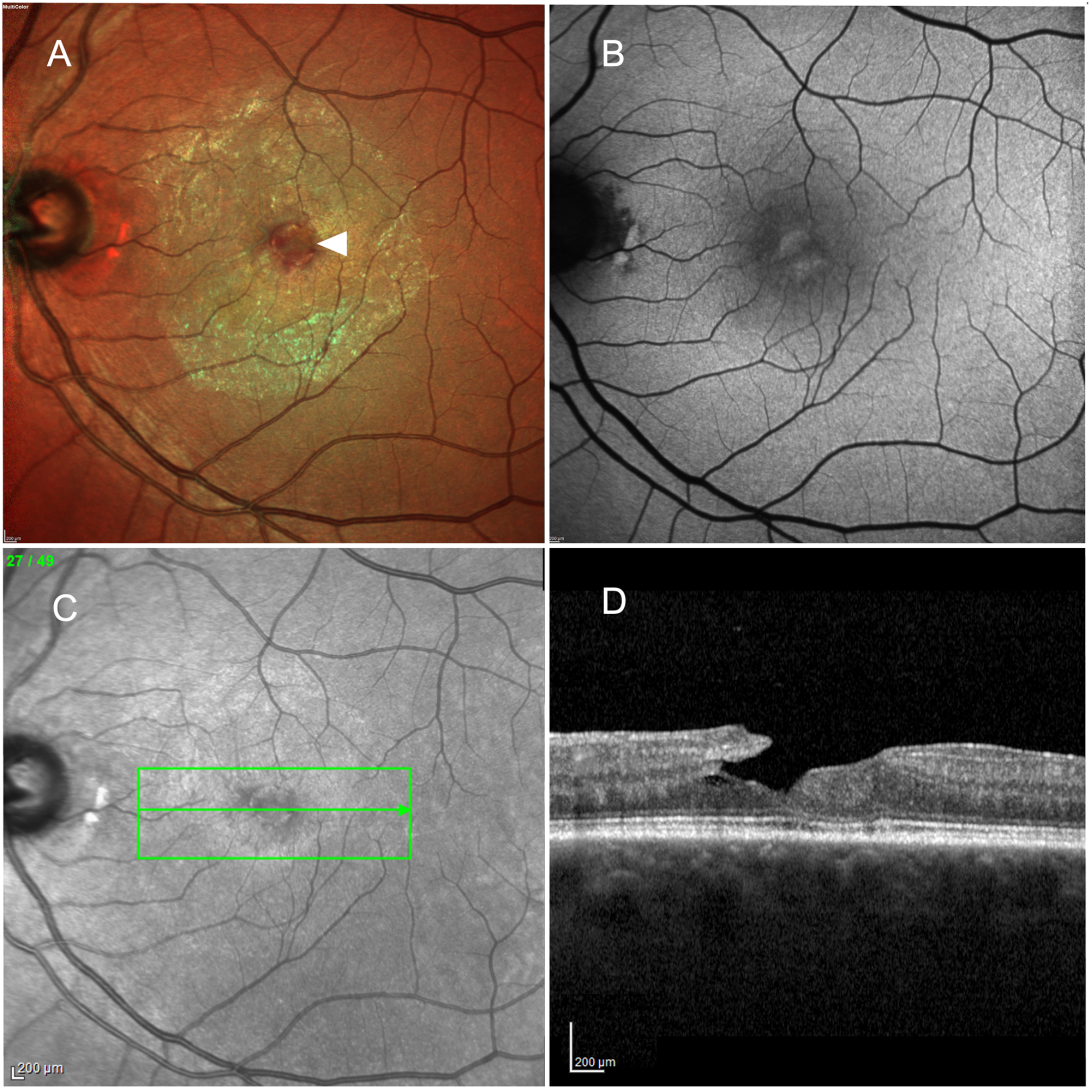
**Multimodal retinal imaging of an eye with LMHs.** (**A**) Multicolor cSLO fundus photograph demonstrates a parafoveal loss of normal foveal reflex with a subtle, irregular foveal contour (*white arrowhead*) and surrounding retinal transparency changes due to extensive epiretinal proliferation. (**B**) FAF shows an abnormal autofluorescence signal characterized by central hyporeflectivity with poorly defined margins, consistent with inner foveal structural alteration. (**C**) En face near-infrared image illustrating the location of the OCT B-scan (*green arrow*) traversing the foveal center. (**D**) Corresponding horizontal SD-OCT B-scan through the fovea demonstrates an irregular foveal contour with a lamellar defect characterized by a partial-thickness foveal cavity, undermined edges, and preservation of the outer retinal layers. Hyperreflective material along the inner retinal surface is consistent with epiretinal proliferation. The EZ remains continuous beneath the lamellar defect.

**Figure 2.**
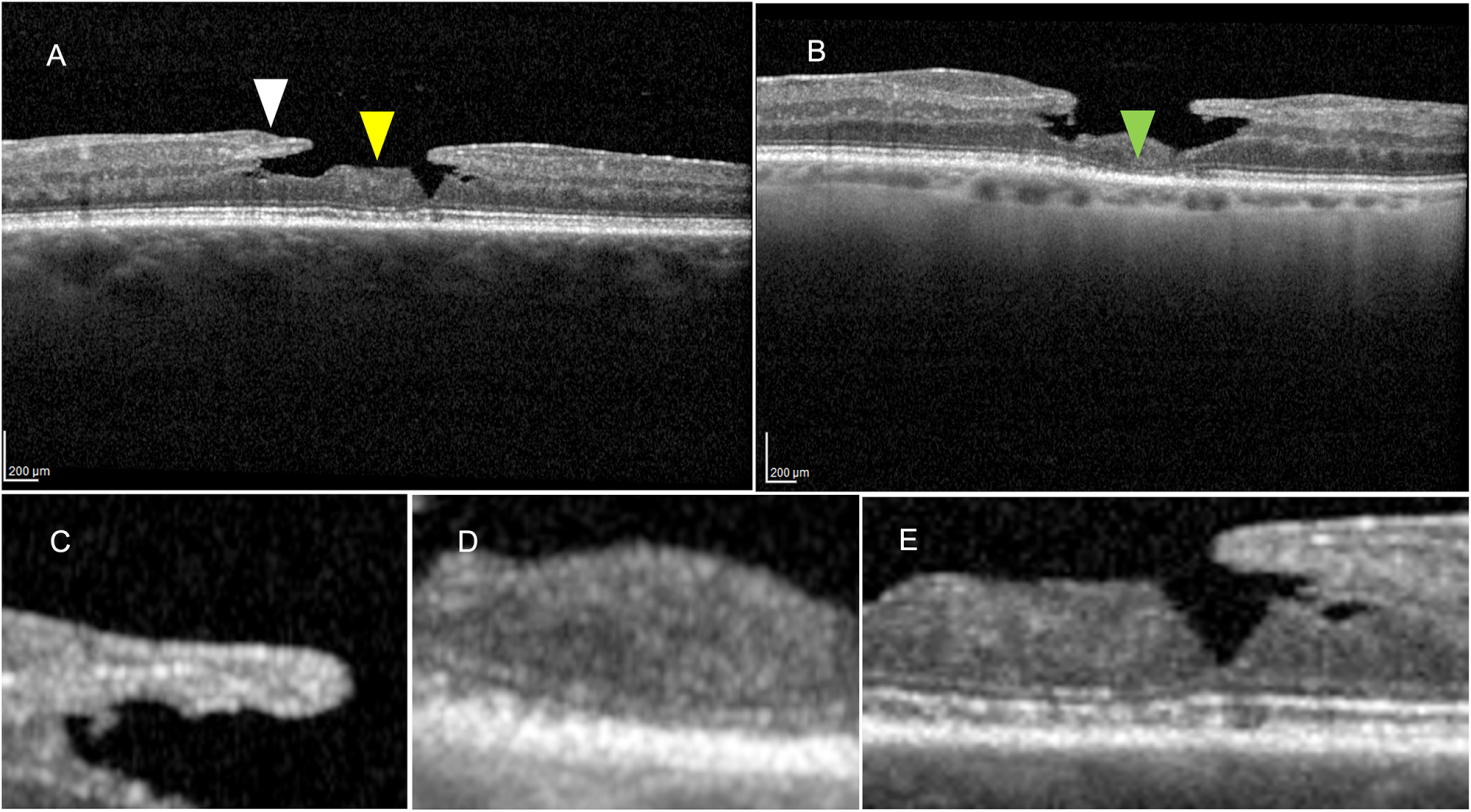
**Representative OCT examples of LMHs with different degrees of foveal thinning reflecting the consensus definition.** (**A**) LMH with CFT ≥ 180 µm showing an irregular foveal contour and a partial-thickness foveal cavity with undermined edges. The *white arrowheads* indicate the epiretinal proliferation, corresponding to the edges of the cavitation and localized loss of foveal tissue. A central mound of residual tissue is shown between the two cavity edges, representing a preserved FB (*yellow arrowhead*). (**B**) LMH with CFT < 180 µm showing a shallower lamellar cavity with relatively preserved CFT. The *green arrowhead* indicates the base of the lamellar cavity, highlighting the point of maximal retinal thinning and EZ defect. (**C**) High-magnification view of EP in case **B**, seen as homogeneous, medium-reflective material adherent to the inner retinal surface. (**D**) High-magnification view illustrating a residual central retinal tissue within the lamellar defect with interrupted EZ. (**E**) High-magnification OCT section of case **A** demonstrating intraretinal cavitation with undermined edges, a structural feature indicating inner retinal tissue loss characteristic of LMH. *Scale bars*: 200 µm.

All SD-OCT scans were independently reviewed by two masked retina specialists experienced in the evaluation of vitreomacular interface disorders (AQ, LC). Each scan was assessed for image quality, correct foveal centration, and accuracy of automated retinal layer segmentation. Scans with a quality score < 20 dB, significant motion artifacts, or extensive segmentation errors were excluded from analysis. Central foveal thickness (CFT) was measured manually on the fovea-centered horizontal B-scan using the built-in caliper tool defined as the vertical distance between the foveal contour and the outer boundary of the retinal pigment epithelium at the foveal center. The central B-scan used for CFT measurement was defined as the scan passing through the center of the lamellar cavity and corresponding to the point of maximal foveal tissue loss. This location was identified as the thinnest neurosensory retinal thickness within the foveal region and confirmed by the scan position on the infrared reflectance image. When multiple scans fulfilled these criteria, the scan showing the maximal lamellar cavity extent was selected. Qualitative OCT features were recorded as categorical variables, including the presence or absence of EP, FBs, and EZ disruption. EZ integrity was evaluated on fovea-centered horizontal B-scans and classified as either intact or disrupted as previously described.^3^ Contralateral fellow eyes were considered healthy and eligible if SD-OCT demonstrated a regular foveal pit configuration with continuous inner retinal layers, preserved outer retinal bands, and no evidence of foveal contour irregularity, cavitation, EP, ERM, or vitreomacular traction (Figs. 1, 2). Discrepancies between graders were resolved by consensus and a final decision by a third senior grader (RM).

### Subgroup Definition Based on CFT

An exploratory subgroup analysis was performed using a CFT threshold of 180 µm. Normative CFT measured by SD-OCT in healthy adult eyes typically ranges from ∼200 to ∼270 µm in large cohorts using current devices, with central point values often > 200 µm.^21^ A value near 180 µm lies substantially below these normal distributions and has previously been described in LMH cohorts as reflective of marked retinal thinning, in addition to the fact that prior OCT literature indicating that CFT values in this range represent substantial thinning relative to normative data reported in healthy eyes corresponding to an estimated 25% to 30% reduction in foveal thickness.^4^^,^^15^^,^^21^ Therefore, we performed the exploratory subgroup analysis using the previously mentioned CFT threshold of 180 µm to differentiate eyes with more advanced structural loss from those with relatively preserved foveal thickness. This threshold was not intended as a universal cutoff or a diagnostic threshold and should be considered exploratory and hypothesis generating.

### Statistical Analysis

A sample size of 48 paired eyes provided 80% power (two-sided α = 0.05) to detect a minimum paired difference in MPOD of approximately 0.04 units, assuming a standard deviation (SD) of the paired inter-eye difference of ∼0.10 (typical for HFP-based MPOD repeatability studies).^9^^,^^22^ Continuous variables were assessed for normality using the D'Agostino–Pearson test. Descriptive statistics are reported as mean ± SD for normally distributed variables. When deviations from normality were detected, logarithmic transformation was applied prior to parametric analyses. To assess measurement reliability, CFT measurements were independently performed by two masked graders. Intergrader agreement was evaluated using the intraclass correlation coefficient (ICC). Categorical variables are reported as frequencies and percentages. Between-eye comparisons of continuous variables between LMH eyes and contralateral healthy fellow eyes were performed using paired two-sample *t*-tests for normally distributed data. Associations between MPOD and continuous clinical or structural variables were evaluated using linear mixed-effects regression models. Variables included in the multivariable model were selected a priori based on biological plausibility and to limit model overfitting given the sample size. Categorical OCT features were included as fixed effects in the regression models. Two-sided *P* < 0.05 was considered statistically significant. Statistical analysis was performed using R 4.2 with the packages lme4 and lmerTest (R Foundation for Statistical Computing, Vienna, Austria).

## Results

**Figure 3.**
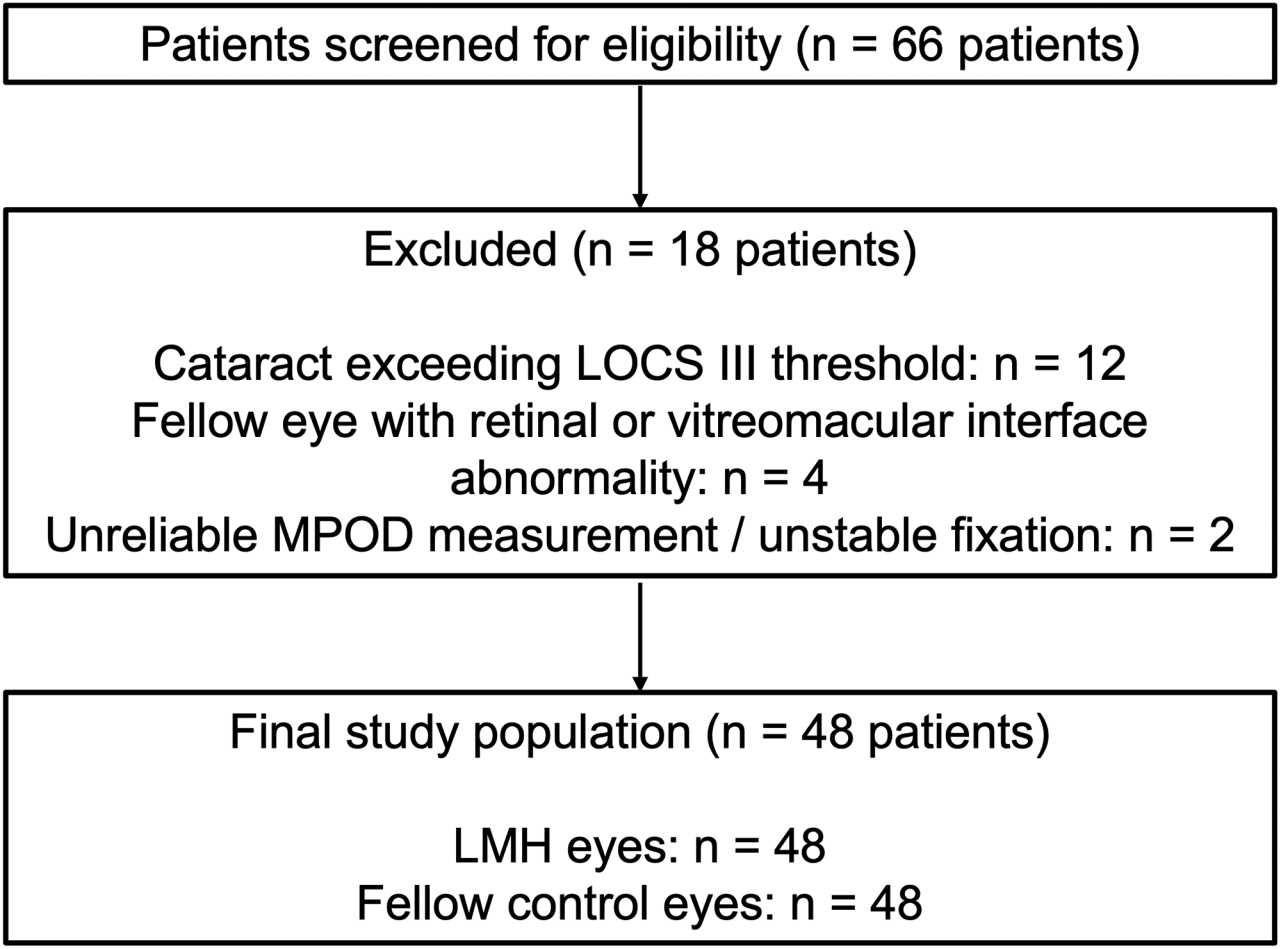
**Flow diagram of patient selection. The flow chart shows the screening and selection of participants included in the study.** A total of 66 patients with unilateral LMH were initially assessed for eligibility. After application of predefined exclusion criteria, including significant lens opacity, retinal or vitreomacular abnormalities in the fellow eye preventing its use as a healthy control, and unreliable MPOD measurements, 48 patients met the inclusion criteria and were included in the final analysis. Both the LMH eyes (*n* = 48) and their contralateral healthy fellow eyes (*n* = 48) were analyzed.

**Figure 4.**
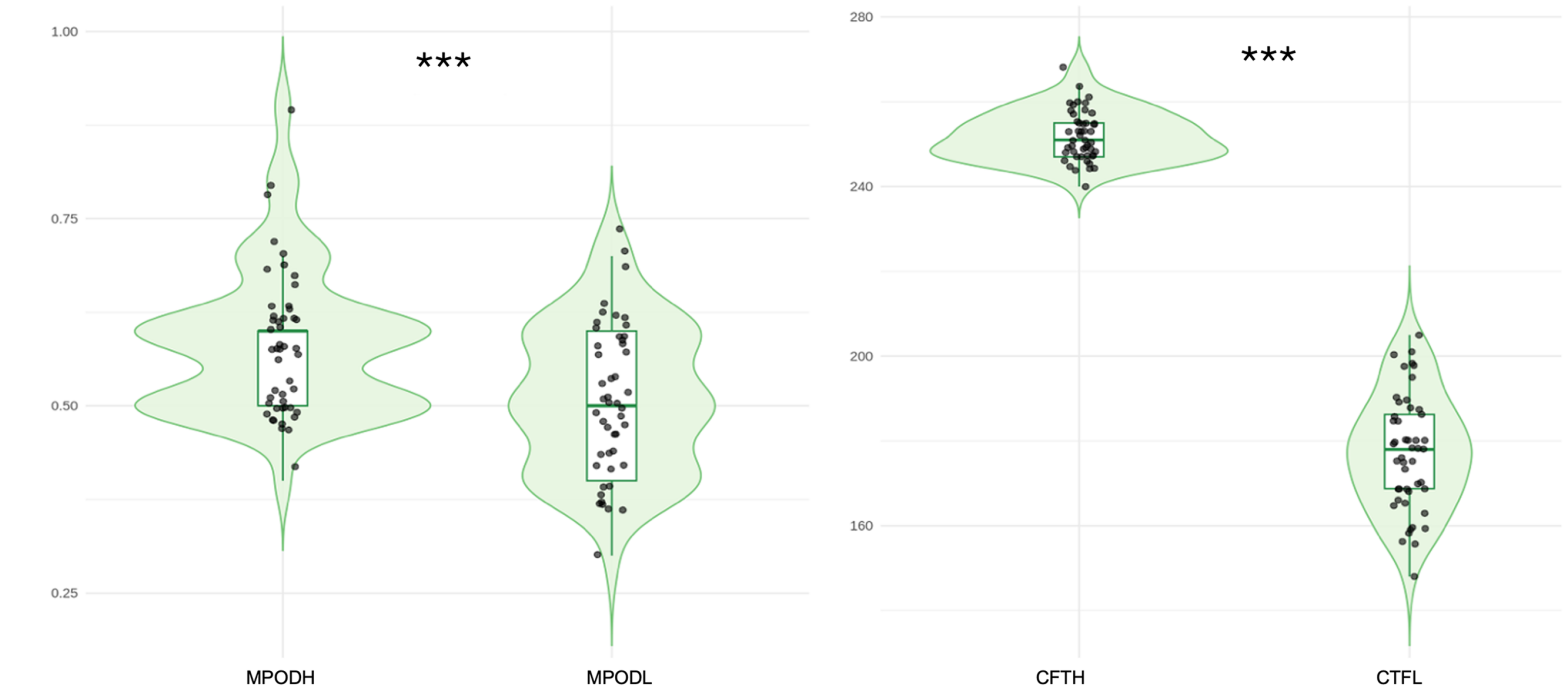
**Paired inter-eye comparison of MPOD and CFT.** Violin plots show the paired inter-eye distributions of MPOD (*left panel*) and CFT (*right panel*) in LMH eyes and contralateral healthy fellow eyes. MPOD and CFT values in fellow eyes (MPODH and CFTH) are shown alongside corresponding measurements in LMH eyes (MPODL and CFTL). Individual data points represent single eyes. Boxplots within each violin indicate the median and interquartile range. Both MPOD and CFT were significantly reduced in LMH eyes compared with fellow eyes. ***P < 0.001.

The final study cohort included 48 patients, each contributing one LMH eye and one contralateral healthy fellow eye, with 18 patients excluded according to the exclusion criteria (Fig. 3). The cohort included 26 women (54.2%) and 22 men (45.8%), with a mean age of 61.7 ± 3.9 years (range, 54–72 years). Descriptive clinical and imaging characteristics are summarized in Table 1. Mean CFT was significantly reduced in LMH eyes compared with fellow eyes (177.2 ± 13.6 µm vs. 251.9 ± 5.9 µm; *P* < 0.0001). Mean MPOD was also significantly lower in LMH eyes (0.508 ± 0.096) than in contralateral healthy eyes (0.583 ± 0.097; *P* < 0.0001). Mean BCVA was worse in LMH eyes (0.28 ± 0.13 logMAR) compared with fellow eyes (0.01 ± 0.03 logMAR; *P* < 0.0001) (Table 1; Fig. 4).

**Table 1.** Inter-Eye Comparison Between LMH Eyes and Fellow Eyes

Variable	LMH Eyes (Mean ± SD)	Fellow Eyes (Mean ± SD)	*P*
CFT (µm)	177.2 ± 13.6	251.9 ± 5.9	<0.0001
MPOD	0.508 ± 0.096	0.583 ± 0.097	<0.0001
BCVA (logMAR)	0.28 ± 0.13	0.01 ± 0.03	<0.0001

Paired inter-eye comparison of CFT, MPOD, and BCVA between LMH eyes and contralateral healthy fellow eyes. *P* values were obtained using paired statistical tests as appropriate.

### Subgroup Analysis by CFT

**Figure 5.**
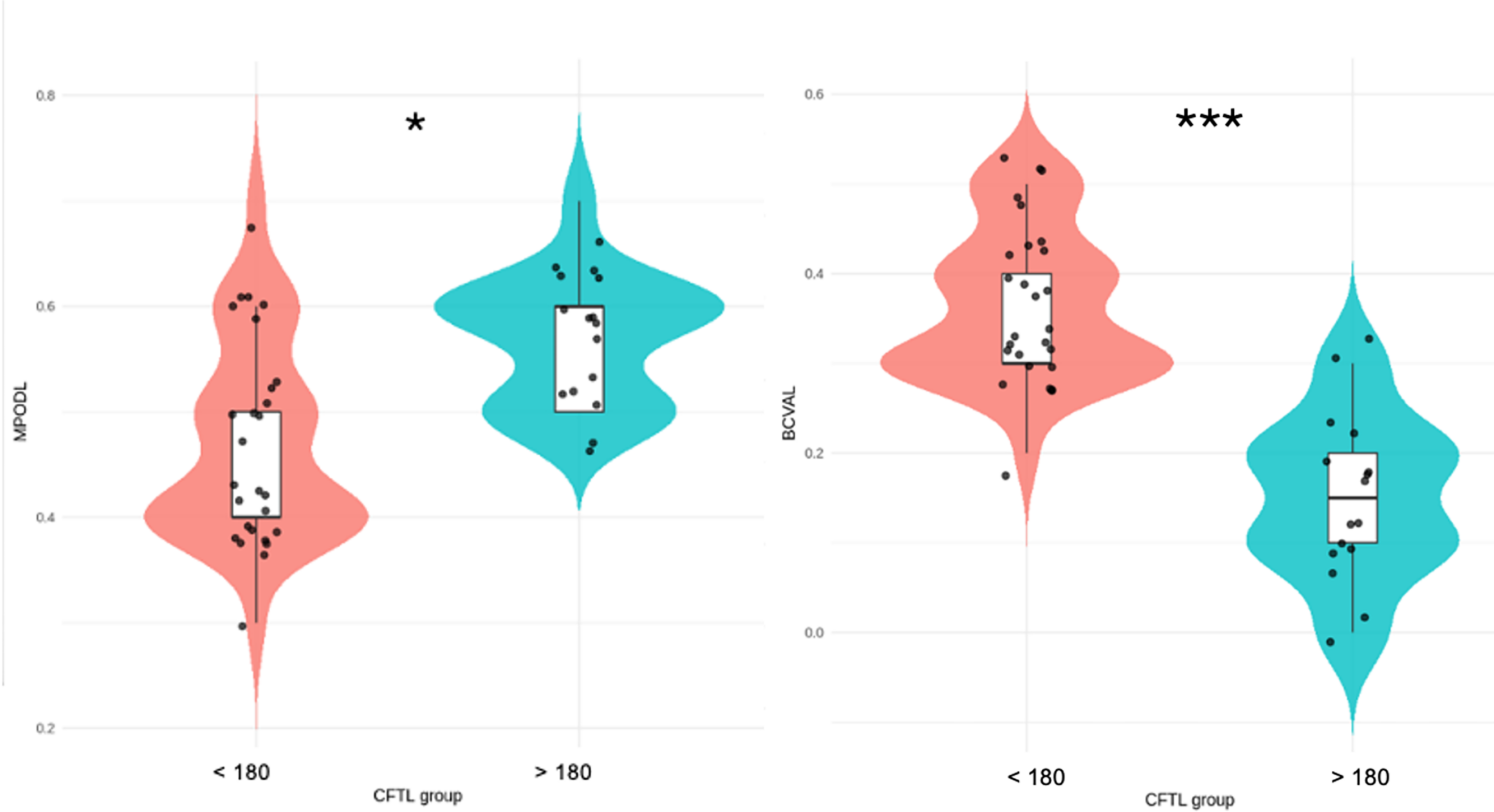
**Distribution of MPOD, CFT, and visual acuity according to CFT subgroup.** Violin plots show the distribution of MPOD (*left panel*), and BCVA (logMAR, *right panel*) in LMH eyes stratified by an exploratory CFT threshold of 180 µm. Eyes with CFT < 180 µm are shown in *red*, and eyes with CFT ≥ 180 µm are shown in *blue*. Individual data points represent single eyes. Boxplots within each violin indicate the median and interquartile range. Eyes with thinner foveas (CFT < 180 µm) demonstrated significantly lower MPOD values, reduced CFT, and worse BCVA compared with eyes with CFT ≥ 180 µm. *P < 0.05, ***P < 0.001.

Using an exploratory CFT threshold of 180 µm, 26 LMH eyes (54.2%) were classified as having CFT < 180 µm and 22 eyes (45.8%) as having CFT ≥ 180 µm. Mean CFT was 167.6 ± 8.1 in eyes with CFT < 180 and 192.6 ± 6.6 in eyes with CFT > 180. Mean MPOD was significantly lower in eyes with CFT < 180 µm compared with those with CFT ≥ 180 µm (0.485 ± 0.095 vs. 0.557 ± 0.076; *P* = 0.0065). Correspondingly, BCVA was worse in eyes with thinner foveas (0.36 ± 0.08 logMAR) than in those with thicker foveas (0.17 ± 0.09 logMAR; *P* < 0.0001) (Table 2; Fig. 5). The ICC for CFT measurements was 0.92 (95% confidence interval [CI], 0.88–0.96).

**Table 2.** Subgroup Analysis of LMH Eyes Stratified by Central Foveal Thickness

Variable	CFT < 180 µm (*n* = 26)	CFT ≥ 180 µm (*n* = 22)	*P*
MPOD, mean ± SD	0.485 ± 0.095	0.557 ± 0.076	0.0065
BCVA (logMAR), mean ± SD	0.36 ± 0.08	0.17 ± 0.09	<0.0001

Comparison of MPOD and BCVA in LMH eyes stratified according to an exploratory CFT threshold of 180 µm. *P* values reflect between-subgroup comparisons.

### OCT Morphologic Features

Among LMH eyes, EP and FBs were present in 45 of 48 eyes (93.8%; *P* < 0.001), and EZ disruption was observed in 6 of 48 eyes (12.5%; *P* < 0.05). None of these features was detected in contralateral healthy eyes.

### Regression Analyses

In linear mixed-effects regression analyses restricted to LMH eyes, MPOD was significantly associated with age (β = −0.010 per year; *P* = 0.0085) and BCVA (β = −0.494 per logMAR; *P* = 0.0207) but not with CFT (*P* = 0.1438). In contralateral healthy eyes, MPOD was significantly associated with age alone (*P* = 0.0002) (Table 3).

**Table 3.** Linear Mixed-Effects Regression Analysis of Factors Associated With MPOD

Predictor	Estimate	SE	95% CI	*P*
Intercept	1.763	0.434	0.89 to 2.64	0.0002
Age (y)	−0.010	0.004	−0.018 to −0.003	0.0085
CFT (µm)	−0.003	0.002	−0.006 to 0.001	0.1438
BCVA (logMAR)	−0.494	0.206	−0.910 to −0.079	0.0207

Results of linear mixed-effects regression models evaluating associations between MPOD and demographic, functional, and structural variables in LMH eyes. Fixed-effect estimates are reported with standard errors, 95% confidence intervals, and *P* values. A random intercept for subject was included to account for within-subject correlation.

## Discussion

In this study, MPOD measured by HFP was significantly reduced in eyes with LMH compared with contralateral healthy fellow eyes. Within LMH eyes, MPOD was associated with BCVA and age, whereas its relationship with CFT became evident only in eyes with more advanced foveal thinning. These findings suggest that MP changes are linked to tissue loss and may reflect progressive inner retinal remodeling related to MCCs rather than early morphologic changes alone.

MP is composed primarily of the xanthophyll carotenoids lutein and zeaxanthin, which are highly concentrated in the fovea and preferentially localized within Müller cells, particularly the specialized MCCs at the foveal center, and partially in Henle's fiber layer and the outer nuclear layer.^5^^,^^6^^,^^8^^,^^10^ Given this localization, MPOD changes may provide an indirect marker of MCC integrity and spatial organization. The finding that MPOD was significantly lower in LMH eyes compared with fellow eyes suggests that LMH-associated retinal remodeling disrupts normal MP distribution or retention, with a potential partial contribution of Henle's fiber layer and the outer nuclear layer.

The observed reduction in CFT in LMH eyes is consistent with the current OCT-based definition of LMH as a disorder characterized by loss of inner foveal tissue rather than simple mechanical deformation. Following the refined diagnostic criteria proposed by Hubschman et al.,^3^ LMH is now distinguished from tractional ERM-associated foveoschisis by the presence of true tissue loss and foveal cavitation with undermined edges. The marked thinning observed in our cohort supports inclusion of predominantly degenerative LMH phenotypes and provides a structural framework for interpreting changes in MP previously investigated in LMH pathophysiology.^4^^,^^23^

The reduction in MPOD observed in LMH eyes is biologically plausible given current understanding of LMH pathogenesis and MCC involvement.^4^^,^^15^^,^^23^ Contemporary OCT-based definitions restrict LMH to lesions characterized by true inner retinal tissue loss, with EP, FBs, and EZ disruption considered supportive features.^3^ Histopathology and immunohistochemical studies have demonstrated that EP is composed predominantly of Müller cell–derived glial elements and frequently exhibits a yellowish appearance, leading to the hypothesis that xanthophyll pigments may be incorporated within this tissue.^24^^–^^26^

Previous investigations of MPOD in vitreomacular interface disorders suggest that MP behavior differs substantially according to the underlying mechanical forces and potential degree of MCC involvement.^18^^,^^27^^,^^28^ In idiopathic full-thickness macular holes (MHs), MPOD showed distinct correlation patterns depending on MH border phenotype, likely reflecting partial preservation of MCCs.^27^ In contrast, eyes with idiopathic ERMs typically demonstrate increased preoperative MPOD, proportional to CFT and outer nuclear layer thickness, consistent with centripetal “packing” of MCCs and associated xanthophylls induced by tangential traction.^18^^,^^29^ We previously demonstrated that MPOD generally decreases following ERM peeling with stage-specific dynamics: Postoperative MPOD reduction is greater in advanced ERM stages and correlates variably with changes in visual acuity, CFT, and outer nuclear layer thickness, suggesting redistribution or partial decompaction of MP after traction release rather than true pigment loss.^18^ Against this background, the MPOD reduction observed in LMHs, particularly in eyes with marked foveal thinning, appears more closely aligned with the MH spectrum than with the ERM. Unlike ERMs, LMHs lack sustained centripetal traction and instead are characterized by progressive inner retinal tissue loss and potential centrifugal migration of glial elements into EP.^7^^,^^8^^,^^30^ The absence of a global MPOD increase and the emergence of correlations with visual acuity only in advanced thinning suggest that MPOD changes in LMH primarily reflect MCC disruption and redistribution rather than reversible mechanical compaction. Because MPOD was measured using HFP, which provides a single central estimate of pigment density rather than spatial mapping, such redistribution could manifest as a reduction in centrally measurable MPOD even in the absence of true pigment loss. Spatially resolved imaging techniques may help clarify whether LMH is associated primarily with pigment depletion or redistribution. Taken together, these comparisons reinforce the concept that MPOD is a dynamic, context-dependent biomarker, whose direction and correlates differ among tractional packing (ERM), focal and traumatic tissue dehiscence (MH), and degenerative inner retinal loss (LMH), with MCC integrity serving as a unifying biological substrate.

The observation that the relationship between MPOD and CFT emerged only in eyes with CFT < 180 µm supports a stage-dependent interpretation. In earlier stages of LMH, residual foveal tissue, the presence of FBs, and centripetal displacement of MCC-derived structures may preserve foveal MP levels despite structural abnormalities.^15^^,^^26^ In contrast, in more advanced LMH characterized by marked thinning, progressive inner retinal tissue loss and centrifugal displacement of EP may exceed the spatial sensitivity of HFP, resulting in an apparent reduction of MPOD. This stage-dependent relationship suggests that early LMH may be characterized predominantly by redistribution of MP-containing tissue, whereas more advanced disease involves sufficient inner retinal tissue loss or displacement to produce a measurable reduction in central MPOD. This interpretation is consistent with OCT studies demonstrating that LMH progression is associated with enlargement of the foveal cavity and centrifugal migration of inner retinal tissue, often accompanied by EP.^31^^,^^32^ Moreover, we cannot exclude that a similar behavior could be appreciated in other conditions, as EP was demonstrated to be detectable in additional contexts.^33^^–^^35^

The association between MPOD and visual acuity further supports the functional relevance of these structural changes. Although photoreceptor integrity (EZ continuity) remains a primary determinant of visual function in LMH, inner retinal alterations may contribute indirectly by disrupting MCC-mediated metabolic and optical support to the fovea.^7^^,^^10^^,^^16^ The independent relationship between lower MPOD and worse BCVA observed in our multivariable analysis suggests that MP changes may capture aspects of foveal dysfunction not fully reflected by thickness measurements alone.

Aging was also independently associated with reduced MPOD in LMH eyes, in line with population-based studies demonstrating age-related declines in MP levels in healthy individuals.^9^^,^^36^ The persistence of this association within LMH eyes indicates that disease-related pigment changes occur on top of normal age-related variation, underscoring the importance of using paired fellow eyes as internal controls.

These findings may be viewed in parallel with other macular disorders characterized by Müller cell dysfunction, such as macular telangiectasia type 2 (MacTel), in which early and spatially selective loss of MP precedes overt photoreceptor degeneration.^37^^,^^38^ Importantly, histologic and imaging evidence suggests that this pigment loss reflects dysfunction or depletion of Müller cells responsible for MP storage and transport rather than primary photoreceptor disease.^10^^,^^16^^,^^38^ Although LMH and MacTel differ substantially in clinical presentation and disease course, both conditions support the concept that MP alterations are sensitive indicators of diseased MCCs.

The functional relevance of these structural and pigment-related changes is supported by the association between reduced MPOD and worse visual acuity in LMH eyes. Although EZ disruption was present in only a minority of cases, it was strongly associated with visual impairment, in agreement with previous reports highlighting the importance of photoreceptor integrity for visual function in LMH.^2^ Notably, in MacTel, reduced central MP has been interpreted as a biomarker of Müller cell compromise and has been shown to precede functional decline.^39^^,^^40^ By analogy, MPOD changes in LMH may similarly serve as an indirect indicator of MCC remodeling and inner retinal integrity. Although the present study does not allow direct comparison between these entities, the observed parallels support the concept that MP alterations are not disease specific but may represent a common downstream consequence of MCC dysfunction across distinct macular disorders. The association between MPOD and BCVA may therefore reflect combined effects of inner retinal remodeling, MCC dysfunction, and subtle photoreceptor compromise that become more apparent in advanced disease. This hypothesis warrants further investigation using spatially resolved MP imaging and longitudinal designs to determine whether MP alterations in LMH follow trajectories analogous to those described in MacTel and whether they can provide early insight into disease progression or functional prognosis.

Several limitations should be acknowledged. First, the cross-sectional design precluded assessment of temporal changes in MPOD and their relationship to LMH progression.

Although strict reliability criteria and repeat testing were applied, HFP measurements may still be influenced by fixation stability. The observed association between MPOD and BCVA therefore raises the possibility that reduced performance on the flicker detection task could contribute, at least in part, to lower MPOD estimates in LMH eyes. Moreover, because inner retinal layer thicknesses, outer nuclear layer measurements, and spatially resolved MP imaging were not assessed in the present study, the proposed link between MPOD reduction and MCC remodeling should be interpreted as a biologically plausible hypothesis rather than direct evidence of structural MCC alteration. Moreover, the present study adopted the contemporary OCT-based consensus definition of LMH; however, it should be acknowledged that the classification of LMH remains partially debated. Blue FAF has been proposed as a complementary imaging modality that may support the presence of true foveal tissue loss through increased autofluorescence resulting from reduced MP masking.

Second, MPOD was assessed using a psychophysical method that does not provide spatial localization of MP distribution and could be sensitive to fixation stability, although reliability criteria and repeat testing were applied. Imaging-based approaches such as two-wavelength FAF allow objective and spatially resolved quantification of MPs across the macula and could offer additional insights into potential spatial redistribution of pigment associated with inner retinal remodeling in LMH. Third, the exploratory CFT threshold should not be interpreted as a diagnostic cutoff and requires validation in independent cohorts. Fourth, the high prevalence of EP and FB limited the ability to assess their independent contributions to pigment and functional changes.

Finally, functional assessment was limited to BCVA. Although visual acuity remains a clinically relevant outcome measure, it may not fully capture subtle functional impairment in early disease. Additional functional testing such as microperimetry or contrast sensitivity assessment could provide a more comprehensive evaluation of macular function in future studies.

In summary, this study demonstrated that MPOD is reduced in eyes with OCT-defined LMH and is associated with BCVA, particularly in more advanced disease characterized by marked foveal thinning. These findings are consistent with a model in which LMH progression involves remodeling of the MCC architecture, leading to redistribution and eventual loss of centrally detectable MP. Multimodal MP assessment and longitudinal studies will be essential to clarify the temporal dynamics of MP redistribution and its role in LMH pathophysiology.
